# Large-scale analysis of Arabidopsis transcription reveals a basal co-regulation network

**DOI:** 10.1186/1752-0509-3-86

**Published:** 2009-09-03

**Authors:** Osnat Atias, Benny Chor, Daniel A Chamovitz

**Affiliations:** 1 Department of Plant Sciences, The George S. Wise Faculty of Life Sciences, Tel Aviv University, Tel Aviv 69978, Israel; 2 School of Computer Science, The Raymond and Beverly Sackler Faculty of Exact Sciences, Tel Aviv University, Tel Aviv 69978, Israel

## Abstract

**Background:**

Analyses of gene expression data from microarray experiments has become a central tool for identifying co-regulated, functional gene modules. A crucial aspect of such analysis is the integration of data from different experiments and different laboratories. How to weigh the contribution of different experiments is an important point influencing the final outcomes. We have developed a novel method for this integration, and applied it to genome-wide data from multiple Arabidopsis microarray experiments performed under a variety of experimental conditions. The goal of this study is to identify functional globally co-regulated gene modules in the Arabidopsis genome.

**Results:**

Following the analysis of 21,000 Arabidopsis genes in 43 datasets and about 2 × 10^8 ^gene pairs, we identified a globally co-expressed gene network. We found clusters of globally co-expressed Arabidopsis genes that are enriched for known Gene Ontology annotations. Two types of modules were identified in the regulatory network that differed in their sensitivity to the node-scoring parameter; we further showed these two pertain to general and specialized modules. Some of these modules were further investigated using the *Genevestigator *compendium of microarray experiments. Analyses of smaller subsets of data lead to the identification of condition-specific modules.

**Conclusion:**

Our method for identification of gene clusters allows the integration of diverse microarray experiments from many sources. The analysis reveals that part of the *Arabidopsis *transcriptome is globally co-expressed, and can be further divided into known as well as novel functional gene modules. Our methodology is general enough to apply to any set of microarray experiments, using any scoring function.

## Background

Experimental microarray gene expression data is analyzed by a variety of bioinformatic techniques. In addition to detecting common gene-specific expression patterns, some methods for gene expression analysis are designed to elucidate module- and system-level organization of the transcriptome. One such highly popular method is gene clustering, based on similarity of expression levels. Many different clustering algorithms have been developed for this purpose [1-5]. Clustering of gene expression data serves as a basis for functional annotations of genes, relying on the notion that genes with a similar expression pattern often share a similar function (reviewed in [6]).

The use of networks in computational biology has greatly enhanced analytical capabilities. Methods for inferring network properties can generally be divided into direct vs. module-assisted methods (reviewed in [7]). Direct methods assign properties to nodes, based on prior knowledge of properties of their direct neighbours. In contrast, module-assisted methods first try to identify clusters of nodes that are highly connected to one another, as inferred from the network topology. These clusters are viewed as modules, which are sets of objects that share some biological identity. Thus, any properties attributed to part of the module are assumed to hold for the entire module. Several studies have utilized gene module detection in gene co-expression networks [8-12]. In this type of biological networks, nodes correspond to genes, and edges connect genes that are co-expressed across a certain set of conditions. A highly-interconnected sub-graph in the network corresponds to a set of genes that are highly co-expressed. Such sets of genes can also be defined as a transcriptional module. Several algorithms have been developed for the detection of such sub-graphs in gene co-expression networks, or other network types [5,13-15].

While graph-based algorithms for module detection are generally applied to data from a single microarray experiment, integration from multiple data sources enhances the predictive power of co-expression analysis. Integration can be made across different types of data from a single species. For example, Gunsalus and colleagues [16] integrated three data sets, microarray, protein interaction and phenotypic signatures, to a single network to describe *C. elegans *embryogenesis. In other works, expression data from several species is combined. For example, a study by Stuart and colleagues [17] integrated expression data from *H. sapiens*, *C. elegans*, *D. melanogaster *and *S. cerevisiae *into a single network, and used network visualization methods to detect functional modules.

Another case of data integration is the detection of gene co-expression across different microarray experiments of the same organism. In a study of human transcriptome, Lee and colleagues [18] collected a set of microarray experiments originating in different labs, and analyzed each of those for gene co-expression. Then they integrated the results into a single network, in which gene modules were detected by clustering. A different approach was taken by Yan and colleagues [19], who formulated a procedure to find co-expressed modules that re-occur in a large number of co-expression networks. They, too, applied their method on human data. Integration of co-expression data from different *Arabidopsis *experiments was performed by Wei and colleagues [20]. However, this study concentrated on the analysis of only 1330 metabolic genes. An *Arabidopsis *full genome analysis using a large collection of microarray experiments was performed by Ma and colleagues [21]. In this research the authors used graphical Gaussian models (GGM) for assessing dependencies between expression levels of genes and constructing a gene network whose edges connected any pair of genes whose partial correlation exceeded a certain value. Detected sub-networks were identified as biologically meaningful gene modules.

Here we use a method similar to Lee and colleagues [18] to analyze transcription data of *Arabidopsis thaliana*. Our overriding goal is to provide a comprehensive general map of Arabidopsis transcription modules that is independent of experimental conditions. Towards this end, we analyzed data from 43 microarray experiments, for about 21,000 Arabidopsis genes. Our method principally differs from that of Ma and colleagues [21] in how gene co-expression is determined. We, too, employ the Pearson correlation coefficient as a similarity measure. But we first calculate co-expression within each experiment separately, and not across all microarrays simultaneously. We developed a novel scoring method that integrates co-expression data from different experiments, which is based on the frequency of co-expressed genes in each dataset. We show that our methodology identifies biologically relevant modules, and therefore can serve as a basis for functional annotation of genes and for a better understanding of the *Arabidopsis *transcriptional regulation machinery.

## Results

### Building a Network of Globally Co-Expressed Genes

We have analyzed 43 *Arabidopsis thaliana *microarray experiments, encompassing 857 hybridization samples, performed in a variety of experimental conditions in 37 different labs (Table 1). The expression data was filtered as described in *Materials and Methods *to increase the accuracy of the analysis, resulting in expression measurements for about 21,000 Arabidopsis genes across the 43 datasets. After filtering, each dataset individually contained expression values of about 17,300 genes, in an average of 20 hybridization samples. Within each dataset we calculated the Pearson correlation coefficients between all gene pairs, as a measure of co-expression between the genes, leading to an analysis of about 2 × 10^8 ^gene pairs.

**Table 1:** List of experiments used in the global co-expression analysis

Accession Number	Number of Samples	Experimental Setup	Sampled Tissue	Lab
E-TABM-63	20	Mutant, Loss of Function	Various	Weigel, D
E-NASC-75	12	Mutant, Hormone Response	Seedling	Sakakibara, H
E-NASC-74	12	Mutant	Seedling	Coates, J
E-NASC-31	12	Mutant	Seedling	Hampton, C
E-NASC-29	12	Mutant, Nutrient Stress	Seedling	Greville, K
E-NASC-1	12	Mutant	Seedling	Cornah, J
E-MEXP-449	12	Loss of Function, Radiation Stress	Leaf	Van Breusegem, F
E-MEXP-444	12	Mutant, Radiation Stress	Seedling	Ruberti, I
E-MEXP-1094	12	Over Expression, Pathogen Stress	Leaf	Tang, Y
E-MEXP-547	14	Mutant, Pathogen Stress	Seedling	Felix, G
E-MEXP-300	15	Mutant	Various	Van Lijsebettens, M
E-MEXP-557	16	Mutant, Radiation Stress	Seedling	Ulm, R
E-GEOD-431	16	Mutant, Pathogen Stress	Unknown	Somerville, S
E-ATMX-3	16	Hormone Response, Over Expression	Unknown	Kim, J
E-TABM-21	18	Mutant, Light Conditions	Various	Weigel, D
E-NASC-76	18	Pathogen Stress	Seedling	Dewdney, J
E-NASC-61	18	Nutrient Stress	Various	Hammond, J
E-NASC-20	18	Mutant, Hormone Response	Whole Plant	De Grauwe, L
E-MEXP-265	18	Tissue Comparison	Various	Turner, SR
E-MEXP-550	20	Radiation Stress	Seedling	Ulm, R
E-MEXP-546	21	Mutant, Pathogen Stress	Leaf	Parker, JE
E-NASC-49	22	Light Conditions	Leaf	Smith, S
E-MEXP-475	23	Hormone Response, Nutrient Stress	Seedling	Bevan, MW
E-MEXP-791	24	Nutrient Stress	Various	Thibaud, MC
E-MEXP-739	24	Pathogen Stress	Leaf	Dudler, R
E-MEXP-509	24	Pathogen Stress	Leaf	Yang, C
E-NASC-77	27	Tissue Comparison	Root	Birnbaum, K
E-MEXP-728	28	Light Conditions, Temperature Conditions, Time Course	Shoot Apex	Weigel, D
E-MEXP-1138	28	Mutant	Pollen	Munster, T
E-MEXP-828	34	Nutrient Stress	Root	Coruzzi, GM
E-TABM-19	36	Mutant	Apex	Weigel, D
E-TABM-18	55	Ecotype Comparison	Seedling	Weigel, D
E-GEOD-911	12	Chemical Stress, Fusion Protein, Over Expression	Seedling	Wagner, D
E-GEOD-3454	12	Mutant, Hormone Response	Seedling	Zheng, ZL
E-GEOD-2848	12	Mutant, Time Course	Flower	Reed, JW
E-GEOD-991	14	Loss of Function, Gain of Function	Seedling	Bergmann, D
E-GEOD-3350	14	Mutant, Hormone Response	Root	Beeckman, T
E-GEOD-3326	16	Mutant, Temperature Conditions	Seedling	Zhu, JK
E-GEOD-3416	18	Light Conditions	Leaf	Stitt, M
E-GEOD-1110	22	Hormone Response	Seedling	Town, CD
E-GEOD-3220	24	Mutant, Pathogen Stress	Leaf	Somerville, S
E-GEOD-4733	27	Mutant, Hormone Response	Stamen	Browse, J
E-GEOD-3709	37	Temperature Conditions, Chemical Stress, Hormone Response	Cell culture	Whelan, J

(*) The accession number refers to the corresponding ArrayExpress repository ID.

Given the co-expression data from all 43 datasets, our goal was to integrate this information into a single network. We found that many gene pairs appear simultaneously only in a small number of datasets (Additional data file 1). To ensure that our analysis will detect genes that are co-expressed across a large variety of conditions, we took into account only gene pairs that appear simultaneously in at least 20 out of the 43 experiments. This threshold allowed for about 50% of all the gene pairs to be considered for further analysis.

We also considered how to weigh the contribution of different datasets to co-expression. The difference in dataset sample size is accounted for by the p-value assigned to the correlation coefficients, as smaller datasets would require a higher correlation coefficient between a pair of genes to match the p-value of lower coefficients in big datasets. However, we found another significant difference between datasets: the percentage of significantly correlated gene pairs is highly variable (see Methods below and figures therein). We argue that it is necessary to compensate for this difference, and therefore devised a suitable scoring function, used to integrate co-expression data from multiple datasets and produce a score for each gene pair, signifying how well the two genes are co-expressed across all datasets. A further discussion of the scoring function and the reasons it was chosen are available in the Methods section.

Scores assigned to the chosen gene pairs were in turn used to build networks, in which nodes represent Arabidopsis genes, and an edge in the network connects any two genes whose score exceeds a given threshold, denoted by *t*_*score*_. These networks were searched for clusters of highly intra-connected nodes using the MCODE algorithm [13]. The resulting clusters are candidates for functional gene modules that share a common expression regulation across the datasets we have used.

**Figure 1:**
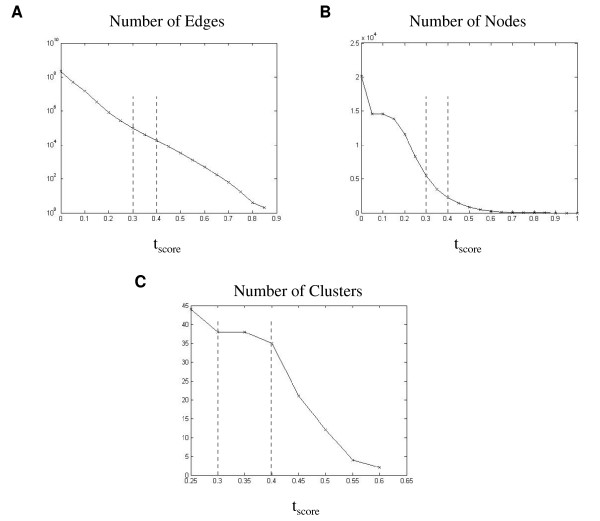
**Affects of score thresholds on the co-expression networks**. Different score thresholds were used to construct networks as described in the text. For each network tested, several parameters were tested: (A) Number of edges, plotted on a logarithmic scale, (B) Number of nodes, (C) Number of clusters found by the MCODE algorithm. The dashed lines mark the chosen 0.3 and 0.4 thresholds chosen.

**Figure 2:**
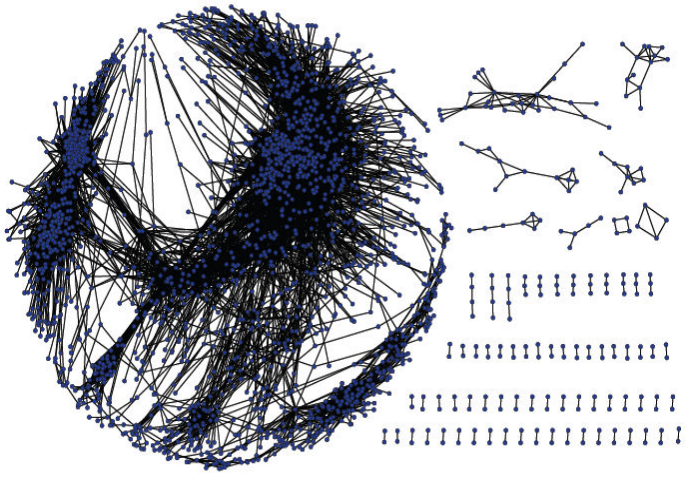
**Networks of globally co-expressed genes**. The network of globally expressed Arabidopsis genes is shown, where the blue nodes are genes and edges connect genes with a co-expression t_score _threshold of 0.4. The network is comprised of a highly interconnected network on the left and isolated sub-networks on the right. A network based on the t_score _threshold of 0.3 was too crowded to show.

**Figure 3:**
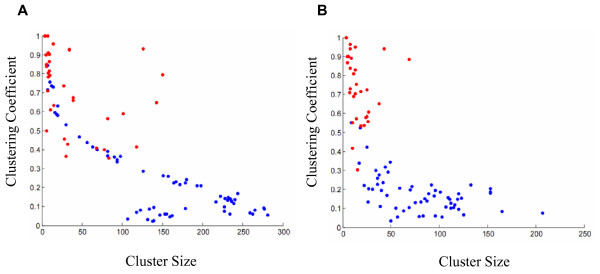
**Comparison between experimental and random networks**. Each cluster found in the 0.3 (A) or 0.4 (B) networks was plotted as a dot according to cluster size and clustering coefficient. Red dots represent clusters in the experimental networks. Blue dots represent clusters found in 10 different random networks, created by random shuffling of edges in the experimental networks.

To select a threshold for *t*_*score*_, we took into account network size, as well as the number of clusters detected by MCODE for different threshold values (Figure 1). We chose to further explore the networks built using the thresholds 0.3 and 0.4 as a good compromise between compact network size and a relatively large number of clusters (Figure 2 and Table 2). We compared these results to those obtained from randomized networks, produced by two different methods, as follows. First, scores calculated for each gene pair were randomly shuffled, and a new network was built using either the 0.3 or the 0.4 thresholds. This procedure was repeated 10 times, and *no clusters *were detected by MCODE in these randomized networks, indicating that the scores calculated using our method represent meaningful interactions between the genes. As a second verification, we randomly shuffled the edges of the 0.3 and of the 0.4 networks, which were created using the correct scores. This procedure was independently repeated 10 times for each threshold, and each random network was searched for clusters using MCODE. Results summarized in Table 3 show that the shuffled random networks have significantly fewer clusters than the real ones. Average cluster size in the random networks was 4-5 times larger than that of the real network, which tended to be smaller and denser than those detected in the random networks (Figure 3). This indicates that the experimental networks have a rich topology, which may represent biological meaning.

**Table 2:** Properties of the networks constructed for the analysis

Network t_score _threshold	Number of nodes	Number of Edges	Number of clusters
0.3	5438	98984	38
0.4	2212	17788	35

**Table 3:** Comparison of clusters found in experimental and randomly generated networks

Threshold	Network Type	Number of clusters (*)	Average cluster size (*)
0.3	Experimental	38	36
	Random	7 (6, 8)	147 (120, 161)
0.4	Experimental	35	16
	Random	6 (5, 7)	83 (63, 112)

(*) For "random", results show the average number of clusters, or cluster size, found in 10 random networks, with the minimal and maximal results shown in parenthesis.

To verify that our scoring system and threshold selection indeed produce gene pairs that are co-expressed across a large number of conditions, we further analyzed the edges that appear in the 0.3 and 0.4 networks. For each edge that connects a pair of genes in the network, we calculated the number of datasets, out of the 43 possible, in which the two genes are significantly co-expressed. On average, each edge in the networks was detected in 20-25 datasets (Additional data file 2), indicating that high scoring edges indeed integrate co-expression data from multiple experiments.

We provide lists of the genes and edges in the networks built using the 0.3 and 0.4 thresholds (Additional data files 3, 4 and 5).

### GO Enrichment Analysis of Network Clusters

To test the biological significance of the two networks, we checked for enrichment of gene ontology (GO) annotations in the clusters found (Tables 4 and 5). 58% of the clusters in the 0.3 network and 60% of the clusters in the 0.4 network had some degree of GO enrichment. Both of these networks contained large clusters, highly enriched in ribosomal or chloroplast genes, as can be expected when searching for modules of globally co-expressed plant genes. Additional enriched clusters appeared in both networks, for example the proteasome core, glycoside biosynthesis, stress response, and more. The two networks are neither identical nor redundant, meaning that some enriched clusters appear in one but not the other, for example, "nucleotide binding" in the 0.3 network, and "response to auxin" in the 0.4 network. Therefore, we continued our investigation with both networks.

**Table 4:** GO Enrichment of clusters found in the 0.3 co-expression network

Network t_score _threshold	Cluster ID	Cluster Size	Enriched GO Terms	Percent of Genes with Enriched GO Terms
0.3	1	126	ribosome	91.26%
	2	150	chloroplast; photosynthesis; structural constituent of ribosome	73.33%; 23.33%; 8.66%
	3	143	plastid; ribosome	48.25%; 13.98%
	4	34	None	None
	5	34	nucleotide binding	32.35%
	6	39	ribonucleoprotein complex; protein targeting to mitochondrion	51.28%; 10.25%
	7	14	None	None
	8	118	plastid; organelle lumen	48.3%; 9.32%
	9	101	plastid	32.67%
	10	68	plastid	45.58%
	11	39	glycoside biosynthesis	10.25%
	12	27	None	None
	13	83	plastid; plastid organization and biogenesis	63.85%; 6.02%
	14	82	plastid; mitocohndrion; response to stress; ER; response to temperature stimulus	32.92%; 17.07%; 14.63%; 13.41%; 8.53%
	15	9	None	None
	16	32	None	None
	17	78	plastid; isoprenoid biosynthesis; carotenoid biosynthesis	44.87%; 7.69%; 5.12%
	18	8	phenylpropanoid metabolic process; flavonoid biosynthesis; response to UV	75%; 62.5%; 50%
	19	7	proteasome core complex	100%
	20	10	None	None
	21	9	response to stress; defense response	55.55%; 44.44%
	22	30	plastid	66.66%
	23	11	None	None
	24	8	None	None
	25	28	plastid; cellular carbohydrate metabolism	39.28%; 21.42%
	26	9	None	None
	27	6	DNA metabolic process; nucleosome assembly	100%; 83.33%
	28	6	None	None
	29	8	None	None
	30	7	mitochondrion	71.42%
	31	6	ATP Binding; response to heat	66.66%; 66.66%
	32	5	None	None
	33	15	response to heat	46.66%
	34	6	None	None
	35	4	None	None
	36	4	None	None
	37	4	None	None
	38	4	structural constituent of cell wall	100%

**Table 5:** GO Enrichment of clusters found in the 0.4 co-expression network

Network t_score _threshold	Cluster ID	Cluster Size	Enriched GO Terms	Percent of Genes with Enriched GO Terms
0.4	1	69	ribonucleoprotein complex	94.2%
	2	43	chloroplast; photosynthesis	95.34%; 62.79%
	3	25	mitochondrial part	16%
	4	38	plastid; structural constituent of ribosome; peptydil-prolil cis-trans isoemerase activity	68.42%; 23.68%; 10.52%
	5	13	None	None
	6	25	structural constituent of ribosome	84%
	7	27	chloroplast; photosynthesis	74.04%; 14.81%
	8	14	None	None
	9	19	nuclear part	26.31%
	10	13	plastid; photosynthesis	84.61%; 30.76%
	11	8	ER	87.5%
	12	13	chloroplast; plastid	76.92%; 61.53%
	13	8	glycoside biosynthetic process	50%
	14	14	None	None
	15	8	None	None
	16	24	chloroplast; plastid	66.66%; 41.66%
	17	11	chloroplast	72.72%
	18	26	chloroplast; ribosome	65.38%; 26.92%
	19	19	structural constituent of ribosome	89.47%
	20	9	None	None
	21	22	chloroplast; oxidoreductase activity; structural constituent of ribosome	45.45%; 40.9%; 31.81%
	22	6	None	None
	23	7	None	None
	24	11	None	None
	25	5	structural constituent of cell wall; cellulose and pectin-containing cell wall organization and biogenesis	100%; 100%
	26	5	None	None
	27	8	None	None
	28	10	structural constituent of ribosome	80%
	29	15	ribosome biogenesis and assembly	33.33%
	30	5	None	None
	31	10	None	None
	32	4	response to auxin stimulus	100%
	33	4	None	None
	34	4	nucleosome assembly	100%
	35	4	None	None

Close or overlapping GO terms were not listed in the table. A complete list is available in Additional data file 7. Corrected p-values for all GO enrichments are lower than 0.002. Comment applies to both Table 4 and Table 5.

### Investigating the Gene Clusters Using the MCODE Algorithm

**Figure 4:**
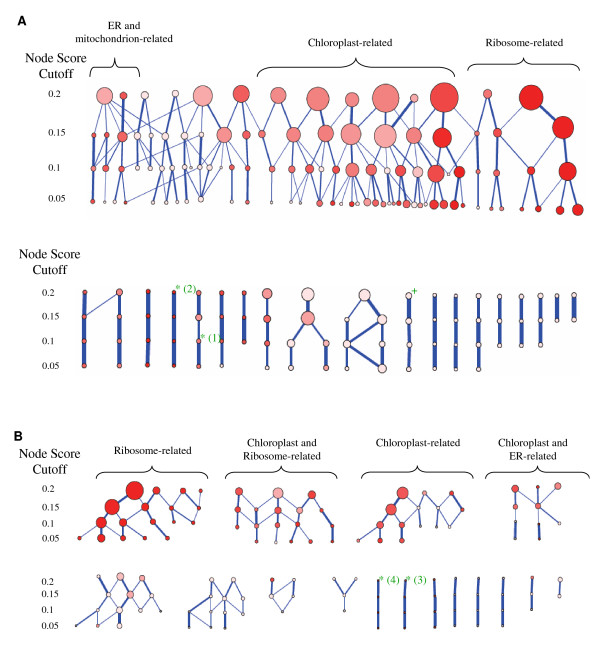
**Genes clusters found using MCODE**. Clusters found using MCODE are visualized as nodes arranged in four levels of decreasing *node score cutoff *(0.2-0.05) as a parameter for MCODE. Node size corresponds to the number of genes in the cluster. Overlapping clusters (that share genes) are connected by an edge, with edge thickness corresponding to overlap size, with the thickest lines indicating that 100% of the child cluster is present in the parent cluster. Node colour intensity corresponds to GO enrichment. Clusters that have no GO enrichment are brightest, while red clusters have close to 100% of the genes sharing an enriched GO term. For clusters with more then one enriched GO term, color intensity shows the percent of genes having the most abundant term. A green asterisk appears above GO-enriched clusters that were used for further analysis. The number besides the asterisk corresponds to the cluster number given in Tables 4 and 5, and in Figure 5. A green plus sign appears above a non GO-enriched cluster that is assigned a putative cell cycle regulation role (see Results and Figure 6).

The cluster detection algorithm used, MCODE, relies on a major parameter called the "node score cutoff", which influences size of the detected clusters and their intra-connectivity. In our initial analysis we have used the default "node score cutoff" value of 0.2 for both the 0.3 and 0.4 networks. However, many of the large clusters in the networks are enriched for multiple and/or general GO terms (Tables 4 and 5), indicating that these clusters are not homogeneous. We suspected that this result would change for different "node score cutoff" values, so we repeated the analysis described above, including testing for enriched GO terms, for decreasing "node score cutoff" values (i.e. stricter clustering parameters). Additional data file 6 lists the genes comprising the clusters found with each tested value, in both the 0.3 and 0.4 networks, and Additional data file 7 lists the enriched GO terms for each cluster. The different cutoff values produce different clusters, including some with new GO terms. As can be expected, there is still significant overlap between clusters found using different cutoff values in the same network. We visualized our results in a hierarchical graph shown in Figure 4, in which nodes represent clusters and each level of the graph shows all clusters found using a particular node score cutoff as a parameter for MCODE. Edges connect overlapping clusters from consecutive levels. When comparing the 0.3 and 0.4 networks, the 0.4 network seems to break into more integral parts, with less overlap between clusters. This is expected, as the 0.4 network is a sub-network of the 0.3 network, containing only those edges representing a higher confidence of co-expression between the genes they connect. Although many of the 0.3 clusters overlap each other, this connectivity still allows for a fairly planar graph, without many intersecting edges. We find that in this near-planar representation, overlapping clusters tend to share similar GO terms (Figure 4A).

Two types of modules that differed in their sensitivity to the node-score cutoff parameter were identified in the regulatory networks. The first type, shown in the top rows in Figure 4A and 4B, is comprised of a large number of clusters that are highly interconnected; clusters found using a less restricted (larger) node score cutoff were generally large, and these clusters broke down into two or more smaller clusters when using a more restricted (lower) node score cutoff. Many of these lower tier modules shared genes with more than one larger precedent cluster. This cluster-instability shows the dynamics of the MCODE algorithm when finding clusters in large, highly connected networks. The bottom rows in the Figure 4A and 4B show the second type of clusters in the networks: in this type, lowering the node score cutoff had little effect of cluster size or stability.

We hypothesized that these two types of clusters found using the same algorithm may be, apart from a property of the network analyzed and of the algorithm itself, a representation of a meaningful biological distinction between the two types of clusters. Specifically, we conjecture that the first type of highly overlapping clusters may be part of larger general regulatory networks, whereas the clusters of the second type represent groups of genes with a specific and specialized regulation pathway.

To test this hypothesis, we checked for the most abundant GO term in each of the clusters in the 0.3 network, and compared between the two types - the interconnected clusters in the top row of Figure 4A, and the more distinct clusters in the bottom row of Figure 4A. Overall, our hypothesis was born out. As a measure of GO term generality, we determined the number of GO term children for the most highly enriched GO term for each cluster. While the average number of children was not statistically different between the two types of clusters (169 for the node-score-dependent group versus 186 for node-score-independent group, p-value of 0.745), the median number of children was statistically different between the two types of clusters (71 for the node-score-dependent group versus 14 for node-score-independent group, p-value of 0.017).

To summarize, we believe that both networks contain valuable information, as the 0.4 clusters reveal more specific gene modules, while the much larger 0.3 network contains more gene modules.

### Investigation of the Results Using Genevestigator

**Figure 5:**
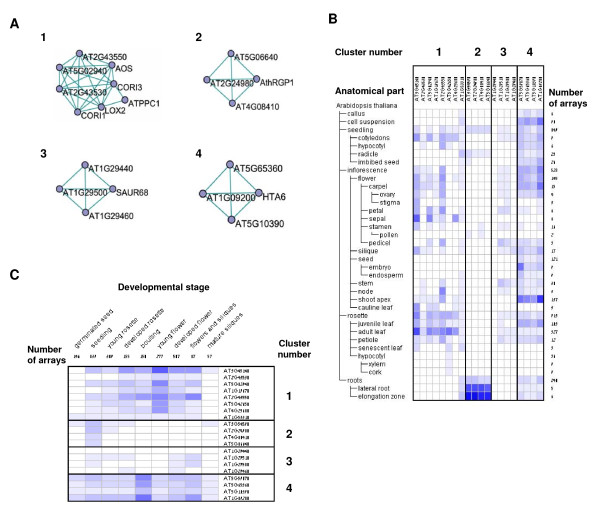
**Analysis of gene modules using Genevestigator**. Expression of four clusters (see Tables 6 and 7, Figure 4) was analyzed using Genevestigator. (A) Graph showing the genes in the clusters and the edges that exist between them in the 0.3 and 0.4 networks (each cluster shows edges from the network it was detected in). Expression according to (B) anatomical tissues or (C) developmental stages, is shown. Expression levels are shown in heat maps, where dark blue indicates maximal expression. Figures in B and C were generated using Genevestigator.

The globally co-expressed gene modules detected in the networks may serve as a basis for more extensive studies of genes and modules of interest. A simple, straightforward analysis can be done using *Genevestigator *[22], a gene expression analysis tool for Arabidopsis and other organisms. Here we show selected examples of some of the analysis we were able to perform using this tool. For the analysis, we have chosen 2788 samples available in the *Genevestigator *database that encompass all high quality experiments performed using the 22k Arabidopsis Affymetrix chip. Experiments already used in our co-expression analysis were excluded from the comparison, to limit bias. Using the *Genevestigator *Analysis tool, we compared the expression levels of the genes in four of our clusters, two from each of the two networks (Figure 5A). These four clusters are all classified as node-score-independent clusters, and were identified as enriched for specific GO terms (Table 6). As seen in Figure 5, the modules we identified contain genes which also appear co-expressed in *Genevestigator*. For example, the genes in these four modules behave as four unique clusters in both the plant anatomy (Figure 5B) and plant development (Figure 5C) analyses. This provides a verification of our results with regard to these clusters, as well as an initial insight into the anatomical and developmental conditions under which the modules are likely to be biologically relevant. For example, the cluster marked as #2 is functional in cell wall structure (see Table 6), and according to the *Genevestigator *data is preferentially expressed in seedling roots.

**Table 6:** GO-Enriched clusters that were analyzed using Genevestigator

Cluster number	Network threshold	Node score cutoff	Cluster ID (*)	Enriched GO terms
1	0.3	0.1	26	defense response
2	0.3	0.2	38	structural constituent of cell wall
3	0.4	0.2	32	response to auxin stimulus
4	0.4	0.2	34	nucleosome assembly

(*) Cluster ID as given in Additional data files 6 and 7.

Table 7 provides more details about the genes appearing in the four clusters. Each of these clusters contains both known as well as putative, not well characterized genes. For example, cluster 1 (defense response) contains a number of genes previously known to be expressed in response to wounding [23,24]. In some cases, clusters appear to contain genes that have undergone duplication, which may explain their co-expression (e.g., genes AT1G29500 and AT1G29510 in cluster 3, response to auxin stimulus). In other cases, further biological tests are required to establish what transcriptional regulation networks connect the clustered genes.

**Table 7:** List of genes appearing in the four GO-Enriched clusters analyzed using Genevestigator

Cluster	Gene ID	Gene name	Description
1	AT2G43530		Encodes a defensin-like (DEFL) family protein.
defense response	AT4G23600	CORI3	Encodes cystine lyase which is expected to be involved in amino acid metabolism, providing the plant with cysteine and the generation of precursors of ethylene biosynthesis. mRNA levels are elevated in response to wounding
	AT5G02940		similar to phosphotransferase-related [Arabidopsis thaliana]
	AT1G53310	ATPPC1	Encodes one of four Arabidopsis phosphoenolpyruvate carboxylase proteins
	AT5G42650	AOS	Encodes a member of the cytochrome p450 CYP74 gene family that functions as an allene oxide synthase.
	AT1G19670	CORI1	Chlorophyllase is the first enzyme involved in chlorophyll degradation.
	AT3G45140	LOX2	Chloroplast lipoxygenase required for wound-induced jasmonic acid accumulation in Arabidopsis. Mutants are resistant to Staphylococcus aureus and accumulate salicylic acid upon infection.
	AT2G43550		Encodes a defensin-like (DEFL) family protein.
2	AT5G06640		proline-rich extensin-like family protein
structural constituent of cell wall	AT3G54590	AthRGP1	Encodes a hydroxyproline-rich glycoprotein
	AT4G08410		proline-rich extensin-like family protein
	AT2G24980		proline-rich extensin-like family protein; Identical to Extensin-2 precursor (EXT2)
3	AT1G29440		auxin-responsive family protein
response to auxin stimulus	AT1G29500		auxin-responsive protein, putative
	AT1G29510	SAUR68	SAUR68 (SMALL AUXIN UPREGULATED 68)
	AT1G29460		auxin-responsive protein, putative
4	AT5G65360		histone H3; Identical to Histone H3.2 (HTR1) [Arabidopsis Thaliana]
nucleosome assembly	AT1G09200		histone H3; Identical to Histone H3.2 (HTR1) [Arabidopsis Thaliana]
	AT5G10390		histone H3; Identical to Histone H3.2 (HTR1) [Arabidopsis Thaliana]
	AT5G59870	HTA6	Encodes HTA6, a histone H2A protein.

### Clusters of Unknown Function

**Figure 6:**
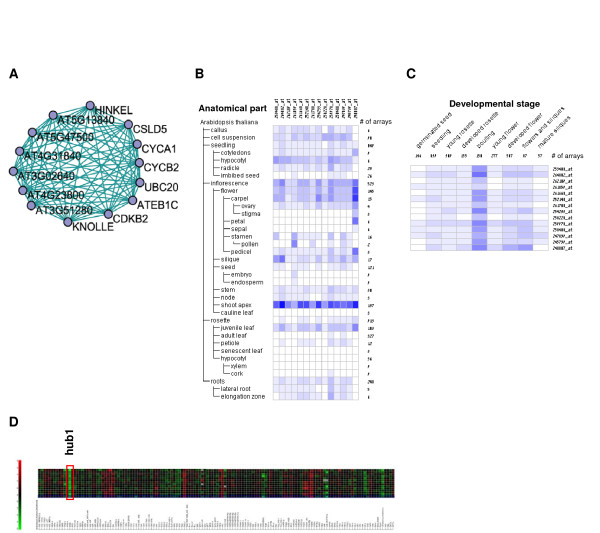
**Genevestigator analysis of a putative cell-cycle regulated cluster**. Cluster 7 from the 0.3 network, detected using the 0.2 node score cutoff, (see gene list at Table 7, Figure 4) was analyzed using Genevestigator. (A) Graph showing the genes in the cluster and the edges that exist between them in the 0.3 network. Expression according to (B) anatomical tissues or (C) developmental stages, is shown. Expression levels in B and C are shown in heat maps, where dark blue indicates maximal expression. (D) Gene expression in different mutants. Expression levels are shown in a heat map in which intense green and red indicate down- or up-regulation in comparison to wild type, respectively. The red rectangle emphasizes the genes expression in the *hub1 *mutant (see text for details). Figures in B, C and D were generated using Genevestigator.

We next examined clusters that had no detected GO enrichment. Careful manual curation of some of these clusters identified new regulation networks. For example, within the list of genes of cluster ID 7 in the 0.3 network using the 0.2 node score cutoff, we noticed several genes that appear to be related to cell cycle control, even though this cluster had no enriched GO term detected (see Table 4 and Table 8 for the list of genes in this cluster). This cluster was found to be co-expressed using the data from *Genevestigator *in different tissues (Figure 6B) and different developmental times (Figure 6C). In the former, high expression of module genes is detected primarily in highly dividing tissues. To further substantiate our hypothesis that this is a cell-cycle regulated module, we also examined the expression of the genes in the cluster in different mutants (Figure 6D). In support of our hypothesis, we found that all the genes in the cluster are highly down-regulated in the *hub1 *mutant. HUB1 (also known as ANG4) is a histone monoubiquitinase, and the *hub1 *mutant has increased cell cycle duration in young leaves [25].

**Table 8:** List of genes appearing in the putative cell cycle regulated cluster

Gene ID	Gene name	Description
AT1G44110	CYCA1	CYCA1;1 (CYCLIN A1;1); cyclin-dependent protein kinase regulator
AT1G08560	KNOLLE	member of SYP11 Gene Family
AT1G18370	HINKEL	Mutant has cytokinesis defects; seedling lethal
AT5G47500		pectinesterase family protein
AT5G67270	ATEB1C	encodes a homolog of animal microtubule-end-binding protein. There are two other members of this family. EB1 forms foci at regions where the minus ends of microtubules are gathered during mitosis and early cytokinesis.
AT4G31840		plastocyanin-like domain-containing protein
AT1G50490	UBC20	Encodes one of two ubiquitin-conjugating enzymes belonging to the E2-C gene family (the other being UBC19). Transcript is always found in diving cells, but also in other non-dividing cells.
AT1G02730	CSLD5	Encodes a gene similar to cellulose synthase. Knock-out mutant has reduced growth, reduced xylan level and reduced xylan synthase activity in stems.
AT1G76540	CDKB2	Encodes a cyclin-dependent protein kinase involved in regulation of the G2/M transition of the mitotic cell cycle.
AT1G76310	CYCB2	core cell cycle genes
AT3G51280		male sterility MS5, putative
AT4G23800		high mobility group (HMG1/2) family protein
AT5G13840		WD-40 repeat family protein
AT3G02640		similar to unknown protein [Arabidopsis thaliana]

### Clusters that are Co-expressed in Specific Conditions

Our analysis was aimed at detecting gene modules that are co-expressed in a wide variety of experimental conditions, therefore we have used a set of diverse microarray experiments for our analysis. However, restricting experimental samples to a certain subset with a common theme may reveal information that is undetected using a variable set of experiments [26]. To check how the experiments used affect the outcome of our analysis, we selected eight experiments out of the 43 used, which were all performed in an experimental setup in which plants were subjected to pathogens. Using only these experiments, we recalculated scores for gene pairs and built new networks using the same method as before, using only gene pairs that appear simultaneously in at least five out of the eight datasets (similar to our threshold of 20 out of 43 datasets used in our previously analysis). Additionally, we chose a t_score _threshold of 0.7 to build the network. We then searched the network for clusters using MCODE default parameters, and analyzed the clusters for enriched GO terms. Tables 9 and 10 show the experiments used for the analysis and the calculated gene clusters, respectively. Additional data file 6 lists the genes in each cluster.

**Table 9:** List of experiments used in the pathogen stress analysis

Accession Number (*)	Number of Samples	Experimental Setup	Sampled Tissue
E-MEXP-1094	12	Over Expression, Pathogen Stress	Leaf
E-MEXP-547	14	Mutant, Pathogen Stress	Seedling
E-GEOD-431	16	Mutant, Pathogen Stress	Unknown
E-NASC-76	18	Pathogen Stress	Seedling
E-MEXP-546	21	Mutant, Pathogen Stress	Leaf
E-MEXP-739	24	Pathogen Stress	Leaf
E-MEXP-509	24	Pathogen Stress	Leaf
E-GEOD-3220	24	Mutant, Pathogen Stress	Leaf

(*) The accession number refers to the corresponding ArrayExpress repository ID.

**Table 10:** GO Enrichment of clusters found in the pathogen stress network

Cluster ID	Cluster Size	Enriched GO Terms	Percent of Genes with Enriched GO Terms	Corrected Pvalue
1	45	protein amino acid phosphorylation; cell communication; defense response; response to biotic stimulus; protein serine/threonine kinase activity; plasma membrane	24.44%; 22.22%; 17.77%; 17.77%; 13.33%; 13.33%	0.005; 0.01; 0.01; 0.002; 0.023; 0.047;
2	72	kinase activity; plasma membrane	20.83%; 11.11%	0.01; 0.017;
3	61	kinase activity	21.31%	0.014;
4	94	plastid; P-P-bond-hydrolysis-driven transmembrane transporter activity; cation-transporting ATPase activity	28.72%; 7.44%; 4.25%	0.002; 0.025; 0.026;
5	117	chloroplast; protein complex; cytosolic part; systemic acquired resistance	31.62%; 17.94%; 5.98%; 4.27%	0.001; 0.001; 0.014; 0.002;
6	83	ribosome; organelle subcompartment; ribosome biogenesis and assembly; photosynthesis	20.48%; 18.07%; 10.84%; 9.63%	0.001; 0.001; 0.002; 0.001;
7	84	plastid; ribosome; cytosolic part	38.09%; 14.28%; 9.52%	0.001; 0.001; 0.002;
8	54	chloroplast; protein complex; photosynthesis; small ribosomal subunit	44.44%; 25.92%; 16.66%; 7.4%	0.001; 0.002; 0.001; 0.029;
9	6	None	None	None
10	25	None	None	None
11	11	structural constituent of ribosome; cytosolic part	81.81%; 54.54%	0.001; 0.001;
12	26	plastid part; organelle membrane	30.76%; 30.76%	0.002; 0.002;
13	6	None	None	None
14	5	None	None	None
15	4	None	None	None

Interestingly, the clusters scored highly by MCODE in the pathogen response network were associated with unique and specific GO terms, such as protein phosphorylation and defense response. This is unlike the general GO terms for modules that had the highest MCODE scores in all the networks that were built using the all 43 experiments. This indicates that using experiments with specific conditions with our analysis methods leads to detection of specific and condition-related gene modules.

**Figure 7:**
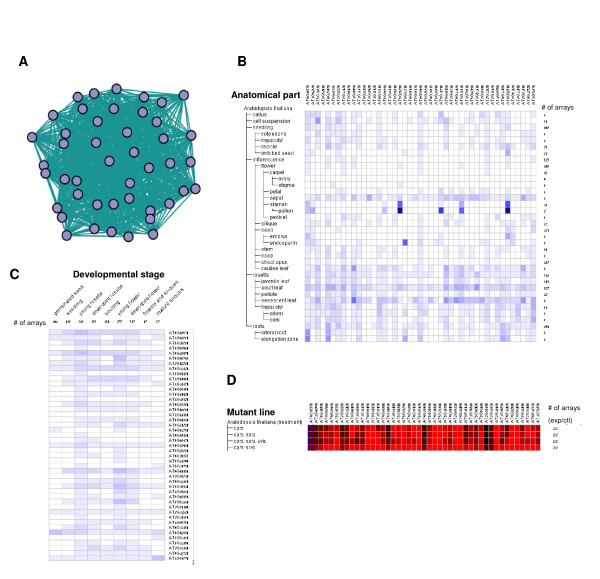
**Genevestigator analysis of a pathogen response cluster from the pathogen response network**. Expression of a cluster found using pathogen stress experiments was analyzed using Genevestigator. (A) Graph showing the genes in the cluster and the edges that exist between them in the pathogen stress network. Expression according to (B) anatomical tissues or (C) developmental stages, is shown. Expression levels are shown in heat maps, where dark blue indicates maximal expression. Expression levels in B and C are shown in heat maps, where dark blue indicates maximal expression. (D) Gene expression in different cpr5 mutants. Expression levels are shown in a heat map in which intense green and red indicate down- or up-regulation in comparison to wild type, respectively. Figures in B, C and D were generated using Genevestigator.

As before, we compared our results for the first cluster detected in the pathogen response network (cluster ID 1) using the *Genevestigator *data. Figures 7B and 7C show that limited overall co-expression is detected within the genes of this cluster. On the other hand, we found that all genes in the cluster are up-regulated in at least one of the *cpr5 *mutant lines (Figure 7D). CPR5 is a known major regulator of pathogenesis-related (PR) genes [27,28], indicating that this cluster is indeed highly specific for pathogen response.

In short, using our method with a specific set of experiments can lead to detection of gene modules that are co-expressed in specific conditions.

## Discussion

### A Method for Detection of Globally Co-regulated Gene Modules

We have presented a general method for detection of gene modules that share a similar regulation pattern across a given set of conditions. The method is based on five main steps: 1) Gathering a set of microarray experiments; 2) Identifying pairs of genes that are significantly co-expressed in individual experiments; 3) Scoring gene pairs for global co-expression, across all experiments; 4) Generating a network of gene co-expression; and 5) Detecting gene clusters. Specific components of the method, such as the scoring function or the cluster-detection algorithm, are easily interchangeable and can be adapted to the task at hand.

While co-expression across a large set of microarray experiments has been previously used in bioinformatic studies [17-20], the method presented here differs from other approaches. As opposed to Stuart [17], Ma [21] and Bergmann [29], and similarly to the works of Lee [18] and Yan [19], we do not calculate co-expression by "concatenating" all hybridization samples from different sources. Instead, co-expression is determined separately per experiment, and the scoring function integrates information from the full data set. As a consequence, experiments with a larger set of hybridization samples do not necessarily have more influence on the result. The approach we have chosen requires the choice of a suitable scoring function, which allows separate treatment of experimental data from different sources. For example, the scoring is independent of the microarray technology used (although we have used data only from Affymetrix chips), and thus data obtained through different technologies could be compared in the same analysis. Along the same lines, our method also allows controlling for differences between experiments from different labs or conditions. For example, we have shown large differences in the proportion of co-expressed genes between different experiments, and could address this issue by adding appropriate weights to the scoring function. Indeed, different labs may produce different results in microarray experiments [30,31], probably due to different lab protocols, analysis methods, and work patterns. Treating each experiment separately and adjusting the scoring function accordingly allows integrating such diverse results. Moreover, using an appropriate scoring function, data can in principle be integrated not just from microarray experiments, but from other sources as well.

Improving the accuracy of the results obtained using this approach may be accomplished in several ways. First, an improved scoring function could be devised that will account for more parameters in the integration of data from different sources. For example, a parameter differentiating between positive and negative co-regulation could be developed (as opposed to our function, which used absolute values and thus did not differentiate between positive and negative co-regulation). Parameters for weighting of experiments to accommodate for experiment-specific factors such as experimental platform, or a parameter for a probabilistic model may also be used to account for random noise in the data set. A second improvement to our method may be obtained by changing the way the network is built. Instead of choosing an arbitrary score threshold, a threshold learning mechanism based on criteria such as coherence of GO term could be incorporated, or an edge-weighted network may be used.

### Specific and Non-Specific Clusters

A few interesting observations are evident by changing the "node score cutoff" parameter of the MCODE algorithm, used to detect gene clusters in our networks. Obviously, changes in this parameter lead to detection of different clusters, demonstrating the high dependence of large-scale data analyses on parameter selection. However, as the "node score cutoff" parameter conveys no direct biological meaning, it may not be necessary to choose a given or solitary value for it. Indeed, our analysis shows that exploration of the parameter space instead provides new insights. In our analyses, it separates between two distinct types of clusters, "unstable", node-score-dependent clusters, whose size and gene composition is highly dependent on this parameter, and "stable" node-score-independent clusters, which retain their size and gene composition across different parameter values. We posit that the division into these two groupings is not merely a consequence of our analysis method, but rather a manifestation of the transcription regulatory pathways that define these clusters. Indeed, GO analyses provided some support for this claim, as the node-score-dependent clusters are enriched for highly general cellular pathways, such as involving the ribosome or chloroplast. Accordingly, their transcriptional regulation is expected to be diverse and highly interconnected. On the other hand, node-score-independent clusters correspond to focused, specific pathways that are likely to be regulated by separate, specific mechanisms.

### Biological Relevance of Our Results

Our method identifies a global co-regulation network, containing thousands of genes, as well as clusters of genes found within the network. The network and component clusters identified represent the minimal gene network that is globally co-expressed in *Arabidopsis*, irrespective of specific growth conditions.

Investigation of the network and the clusters found within it reveals many genes with no or incomplete annotation. Indeed, a large proportion of the *Arabidopsis *genome is under-annotated. The function of such genes can be predicted based on close proximity to well annotated genes in the network. For example, we identified a cluster of 14 cell cycle regulated genes, where only 6 genes are annotated as involved in the cell cycle. Standard GO enrichment analyses were not successful in identifying this function, highlighting the importance of manual curation. That all 14 of these genes are down-regulated in the *hub1 *mutant gave further credence to both our manual curation of the cluster as cell cycle regulated, and to our methodology of network and cluster identification.

Many of the GO-enriched gene clusters in our networks are expected to be globally co-expressed as they pertain to general plant metabolism. These include ribosome, chloroplast and DNA metabolism related clusters. Stress and defense-enriched clusters, and an auxin-response cluster were also identified, emphasizing the importance of these mechanisms in maintaining plant homeostasis. As plant cells are specifically characterized by cell walls, it is also not surprising that a cell wall specific cluster was also identified. Relatively specific modules such as the cell wall, auxin or cell cycle modules are good candidates for further investigation, as they can easily be used to generate specific hypotheses.

More specific modules are most likely to be found by applying our method using different, specific sets of experiments. For example, we detected defense-response specific modules after analyzing a subset of experiments dealing with pathogen stress. Such an approach would extend our results from global transcriptional regulation to tissue-, developmental- or condition-specific networks.

We provide a website [32] holding the co-expression networks and gene modules data, including a gene query interface.

## Conclusion

Using the Arabidopsis genome as a model system, we presented a method for identification of gene modules from diverse microarray experiments. Our method differs from others by the use of a novel scoring function that takes into account the frequency of co-expression in each individual microarray experiment. The analysis reveals that at least a fraction of the *Arabidopsis *transcriptome is globally co-expressed, and can be further divided into functional gene modules. Variation of the parameters employed affects the topology of the networks, allowing for a differentiation between node-score-dependent and node-score-independent modules. By changing the subset of microarray experiments analyzed, condition-specific gene modules are identified as well. This approach is used to provide a comprehensive map of global expression patterns in the *Arabidopsis *transcriptome, including the identification of novel gene modules and assignment of new functions to under-annotated genes. This approach is applicable to any model system.

## Methods

### Data Collection and Filtering

We downloaded Arabidopsis gene expression data from the ArrayExpress website [33]. All experiments available at the time (December 2007), which conformed to the following conditions, were downloaded. First, experiments were required to have statistically processed data available for download, as we wished to avoid analyzing raw data. Second, to minimize the affects of data variability arising from technical issues, we chose a single microarray platform on which all downloaded experiments were performed. The platform chosen was Affymetrix GeneChip Arabidopsis ATH1 Genome array, since it covers most of the Arabidopsis genome (about 24,000 genes, out of about 26,000 known genes), and because it has the largest selection of experiments in the database. Third, as the accuracy of gene expression correlation analysis increases with the number of data points, we used only experiments that contained at least 12 hybridization samples. Out of 61 experiments that passed the above criteria, we manually selected 43 having a complete annotation of the experimental procedures and detailing of statistical methods used for analyzing the raw data. The list of 43 experiments used in our study, including metadata for this set of experiments, is available in Table 1.

We applied mild filtering to the expression data of each downloaded experiment. Probe IDs were converted to gene IDs, using a conversion table built on the basis of the ATH1 array annotation files provided by Affymetrix. We removed single probes that match more than one gene, as well as sets of multiple probes that match a single gene. This filtering reduced the number of probes from 22,810 present on the array, to 20,852 probes that have a one-to-one mapping to Arabidopsis genes. The conversion table constructed is available for download as Additional data file 8. Finally, we reasoned that genes with a low expression value across all samples of a dataset would produce unreliable correlation measurements. Therefore, we calculated for each dataset the lower 25^th ^percentile of the gene expression values of all of its genes and samples, and removed from each dataset genes whose maximal expression across all samples did not exceed this value. About 17,300 genes have passed this criterion in each of the 43 datasets.

### Calculation of Pairwise Correlations

In each dataset, the Pearson correlation coefficient was calculated for all possible gene pairs using the *corr *function in Matlab (version 7.2.0.283). Absent and marginal calls in the gene expression measurements were considered as missing values, therefore different gene pairs had different samples from which a joint correlation can be calculated. To answer this problem we used the *pairwise *option for the *rows *parameter of the *corr *function, which determines, for each gene pair separately, which data points should be used for calculation. Samples for which a missing value appears for any of the two genes in question are not considered when calculating the correlation between the genes. We disregarded correlation coefficients whose calculation was based on fewer than five data points. Since on average each dataset contains expression values for 17,300 genes (see explanation above), correlation coefficients were calculated for more than 1.5 × 10^8 ^gene pairs in each of the 43 datasets. These massive calculations were performed on a 64 GB RAM, four Intel Xeon 2.33 GHz CPU machine, as processing of a single dataset required about 15 GB of main memory.

In addition to calculating the correlation coefficient, the Matlab *corr *function was used to output a p-value for each coefficient, testing the null hypothesis of no correlation against the alternative of a non-zero correlation. Within each dataset, the p-values were corrected for multiple testing using the Benjamini and Hochberg method [34]. Correlation coefficients with a corrected p-value that is lower than 0.05 were considered to be statistically significant.

### Edge Scoring and Network Building

**Figure 8:**
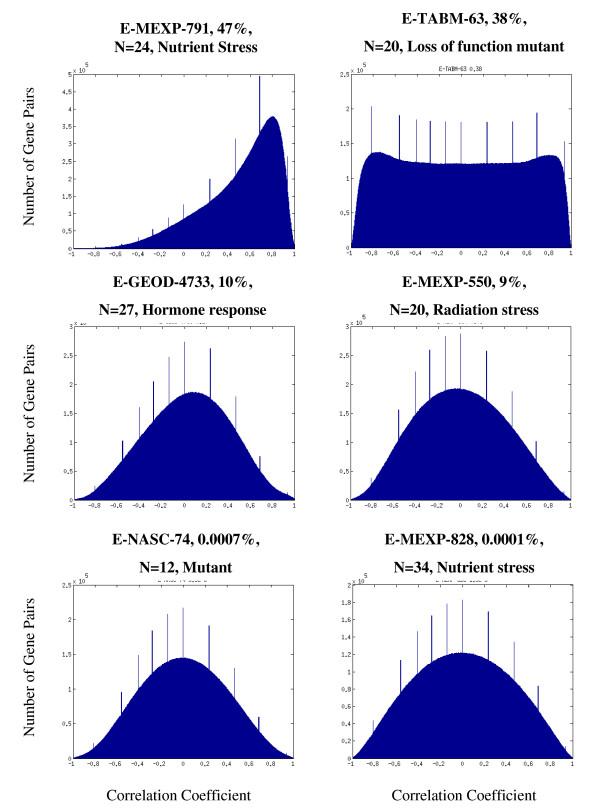
**Distribution of Pearson correlation coefficients in selected datasets**. The two datasets with the highest rate of significant correlation coefficients, two with an average rate and the two with the lowest rate are shown. The ID of the dataset and the percent of significantly correlated gene pairs are shown above each graph. N denotes the number of microarrays used in the experiment and a short description of experimental conditions is included.

**Figure 9:**
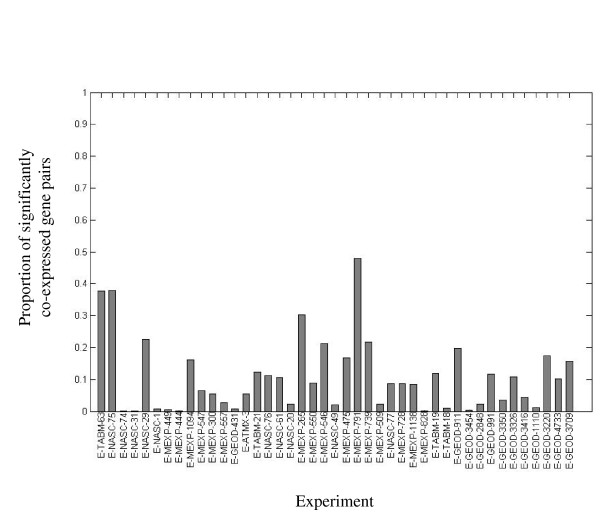
**Percent of significantly co-expressed gene pairs the experiments used**. For each experiment we calculated the number of significantly co-expressed gene pairs that were included in the analysis. The data is presented as a proportion out of all possible gene pairs. Co-expression between a pair of genes is considered as significant if the p-value calculated for the Pearson correlation coefficient is below 0.05.

We sought to integrate the expression correlation data into a network, in which nodes represent genes and edges connect pairs of genes whose expression levels are correlated across a given set of experiments. This raises the question of how to take into consideration different experiments when deciding if an edge should appear in the network. Initially, it may seem appropriate to assign each experiment an equal weight when considering the appearance of an edge. However, we chose not to use this naïve approach, for a number of reasons. First, the number of samples contained in each experiment varies, so correlation coefficients from different datasets cannot be compared directly. To avoid this problem, we did not compare the correlation coefficient themselves, but rather the corrected p-values assigned to each coefficient, as their calculation does incorporate the number of data points used for calculating the coefficients. We considered as statistically significant any correlation coefficient whose corrected p-value was lower than 0.05. Second, the distribution of correlation coefficients in each dataset is often very different from the normal distribution (selected examples are available in Figure 8). Furthermore, the number of statistically significant correlation coefficients observed in different datasets highly varies (Figure 9). For some datasets, out of all correlation coefficients calculated for the dataset, less than 1% are statistically significant, while for others datasets, more than 40% of the correlation coefficients are statistically significant. On average, across all datasets, about 10% of the correlation coefficients are statistically significant. This variation and its possible relation to the underlying biological conditions of each experiment are of interest, and are worth studying in their own right.

Our weighting scheme is inspired by the measure of information of random variables by means of entropy. The more surprising, or unexpected, an event is, the larger is its entropy, and the relative contribution is exactly the log of its inverse probability. In an analog manner, we reasoned that a significant correlation between the expression values of two genes is more informative when it appears in a dataset that has a low rate of significant correlations than in a dataset with a high rate of significant correlations. We therefore devised a function that incorporates this information and computes a score for a pair of genes. A high score signifies that the two genes are highly correlated across a given set of experiments, where experiments with fewer significant correlations are weighted more heavily.

Possibly the most notable difference between our weighted contribution method and the standard, non weighted version, is with respect to "small" datasets - those having few significant correlations. Such datasets will have very little effect on the network produced by an un-weighted method, as very few pairs of genes are taken, and they have to "compete" with larger (more significant correlations) datasets. This would not be a problem if all datasets would give rise to about the same number of significant pairs. However, as pointed out above (Figure 9), this is far from being the case in reality.

Formally, let *D *be a collection of *n *datasets. For each dataset *D*_*k *_in *D*, define *p*_*k *_as the percent of statistically significant correlation coefficients in the dataset. Let <*g*_i_, *g*_*j*_> be a pair of genes, and let *x*_*i*, *j*_^*k *^be an indicator such that *x*_*i*, *j*_^*k *^equals 1 if both *g*_*i *_and *g*_*j *_appear in dataset *D*_*k*_, and 0 otherwise. Due to our filtering, not all datasets have the same genes, but there is a large overlap. Let *y*_*i*, *j*_^*k *^be an indicator of the correlation between *g*_*i *_and *g*_*j *_in dataset *D*_*k*_. Namely, *y*_*i*, *j*_^*k *^equals 1 if *g*_*i *_and *g*_*j *_have a statistically significant correlation (with a corrected p-value of less than 0.05) in *D*_*k*_, and 0 otherwise. Then the score for the pair <*g*_*i*_, *g*_*j*_> over *D *is defined as:

In the nominator, we weight the contribution of *D*_*k *_to the scoring of *g*_*i *_and *g*_*j *_by ln(1/*p*_*k*_). The *lower p*_*k *_is, the *higher *the contribution of the statistically significant correlation observed between *g*_*i *_and *g*_*j *_in dataset *D*_*k*_. Within the set *D*, different datasets may be relevant to different gene pairs, as not all genes appear in all datasets. We therefore normalize the contribution of datasets in which *g*_*i *_and *g*_*j *_are significantly correlated, by the contribution of all datasets in which the two genes appear simultaneously. The denominator is actually the maximal sum that can be achieved for the given gene pair with the set *D*, so the final score is a real number in the range [0, 1].

We remark that the expected value of ln(1/*p*_*k*_) (for a variable taking on value *x*_*k *_with probability *p*_*k*_) equals the *entropy *of that random variable, a central notion in information theory. Entropy-based measurements were used before for analyses of gene expression data [35,36]. In the case where all *p*_*k *_are the same, our measure simply counts the number of datasets where the correlation between *g*_*i *_and *g*_*j *_is significant.

Next, we build a network whose nodes are the 20,852 genes in our datasets. We place an edge between any two genes whose score exceeds a given threshold, which we call *t*_*score*_. This network describes co-expression interactions between gene pairs, based on the set *D *of gene expression datasets used to calculate the scores. In our analysis, we used two sets of datasets (experiments), for each of those the scores are recalculated and a new network is built. As some gene pairs appear simultaneously in only a small number of datasets, we introduced a second threshold called *t*_*datasets*_. An edge that passed the *t*_*score *_threshold is added to the network only when the number of datasets in which the pair appears exceeds the *t*_*datasets *_threshold. In our analysis the effective threshold chosen was 20, meaning that only gene pairs that appeared in almost 50% of our 43 datasets were considered as candidates for globally co-expressed genes.

### Module Finding and GO Enrichment

Our next step was to employ the networks, constructed using different sets of experiments and different threshold values, to find sets of genes that are significantly correlated across different datasets. These sets of genes are candidates for functional gene modules with a common regulatory network, which is active under the experimental conditions of the datasets used for the analysis. Such gene sets would appear as highly intra-connected node clusters in the co-expression networks. We used the MCODE v1.2 plugin in Cytoscape 2.4.1 [13] to detect such clusters, under the following default parameters: No loops included, degree cutoff is 2, haircut is on, no fluff, k-core is 3 and max depth is 100. We have changed the node score cutoff between different runs, as shown in the results section. The clusters outputted by the MCODE algorithm were tested for GO annotation enrichment using the TANGO algorithm in Expander 4.0 [37].

### Robustness Analysis

To validate that the gene sets found by our procedure are meaningful and not random or sporadic, we performed two robustness tests on the network built using all available 43 datasets. First, to check whether the scoring function imposes a network topology that is prone to having spurious intra-connected node clusters, we calculated scores for all gene pairs as explained above, and then randomly shuffled the scores, so that almost all gene pairs were assigned a score that was not originally their own. The procedure for building a network and finding gene clusters was performed as before, using the same thresholds that were used for the experimental network.

Second, to check whether clusters similar to those found in the experimental network appear in a random network with the same degree distribution, a network was built according to the regular procedure, but before searching for gene clusters, the edges were shuffled in a degree-preserving fashion. Shuffling is performed by randomly selecting two edges (*g*_*k*_, *g*_*l*_) and (*g*_*m*_, *g*_*n*_) that do not share any nodes. The selected edges are removed from the graph and the edges (*g*_*k*_, *g*_*m*_) and (*g*_*l*_, *g*_*n*_) are added, as long as the newly added edges do not exist in the graph. This step is repeated for 4 times the number of nodes in the graph.

### Differences between general and specific GO terms

To check for GO term difference between node-score-dependant and node-score-independent clusters we downloaded the full GO ontology, and parsed it in order to find the number of children of each node in the GO acyclic graph. A node in the graph is considered a child of another node if there is a directed path leading from the latter to the former. Each cluster with enriched GO terms was assigned the number of children of its most abundant term. To determine significance of the difference between means of each group of clusters we used a two-tailed t-test. To determine significance of the difference between medians of each group of clusters we used a one-tailed Mann-Whitney test.

## Abbreviations

GGM: Graphical Gaussian Models; GO: Gene Ontology.

## Authors' contributions

OA participated in design of the study, carried it out, and drafted the manuscript. BS conceived and participated in the design and coordination of the computational aspects of the study, and helped to draft the manuscript. DAC initiated the study, participated in its design and coordination, and helped to draft the manuscript. All authors read and approved the final manuscript.

## Supplementary Material

Additional file 1 **Distribution of the number of datasets in which each gene pair appears**. A figure showing a histogram of the number of datasets in which each gene appears. Click here for file

Additional file 2 **Distribution of the number of datasets that contribute to each edge**. A figure showing a histogram of the number of datasets that contribute to each edge in the co-expression network. Click here for file

Additional file 3 **Genes appearing in the co-expression networks**. A list of the genes that appear in the 0.3 and 0.4 co-expression networks that are discussed in the article. Click here for file

Additional file 4 **Edges of the 0.3 network**. A list of the edges appearing in the 0.3 network. Each line lists the gene identifiers of the two nodes connected by an edge. Click here for file

Additional file 5 **Edges of the 0.4 network**. A list of the edges appearing in the 0.4 network. Each line lists the gene identifiers of the two nodes connected by an edge. Click here for file

Additional file 6 **Genes appearing in network clusters**. A table listing the genes that appear in the clusters found in the 0.3, 0.4 and pathogen stress-related co-expression networks. Click here for file

Additional file 7 **GO enrichment of clusters found using different MCODE parameters**. A table listing the enriched GO terms of all clusters found in the analysis, using different score thresholds and cluster detection parameters. Cluster sizes (the number of genes in each cluster) are also listed. Click here for file

Additional file 8 **Probe ID conversion table**. A table mapping Affymetrix probe ids from the ATH1 array to AGI gene id format, provided in a tab-delimited file format. Click here for file
