# Supplementary material for: Large-scale analysis of Arabidopsis transcription reveals a basal co-regulation network
Source: BMC Syst Biol. 2009 Sep 3;3:86. doi: 10.1186/1752-0509-3-86 (PMC2944327; doi:10.1186/1752-0509-3-86)
Supplement: Additional file 1 — Distribution of the number of datasets in which each gene pair appears. A figure showing a histogram of the number of datasets in which each gene appears. [file 1752-0509-3-86-S1.pdf]

Additional data file 1

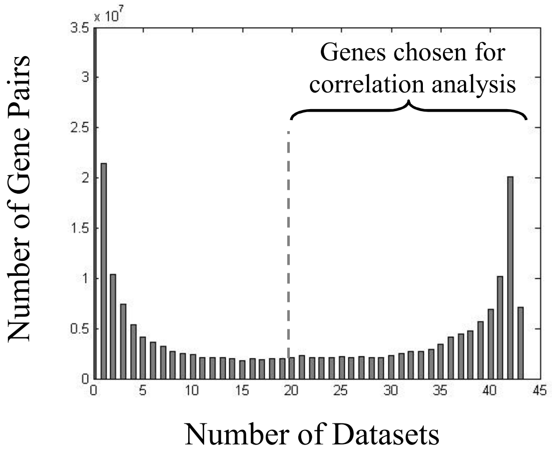

Distribution of the number of datasets in which each gene pair appears
For each gene pair, the number of datasets in which they appear simultaneously was calculated. The data is plotted as a histogram showing the number of gene pairs exist per a specific number of datasets. Only gene pairs that appear in at least 20 datasets (dashed line) were used for further analysis.
