# Supplementary material for: Large-scale analysis of Arabidopsis transcription reveals a basal co-regulation network
Source: BMC Syst Biol. 2009 Sep 3;3:86. doi: 10.1186/1752-0509-3-86 (PMC2944327; doi:10.1186/1752-0509-3-86)
Supplement: Additional file 2 — Distribution of the number of datasets that contribute to each edge. A figure showing a histogram of the number of datasets that contribute to each edge in the co-expression network. [file 1752-0509-3-86-S2.pdf]

Additional data file 2

A B

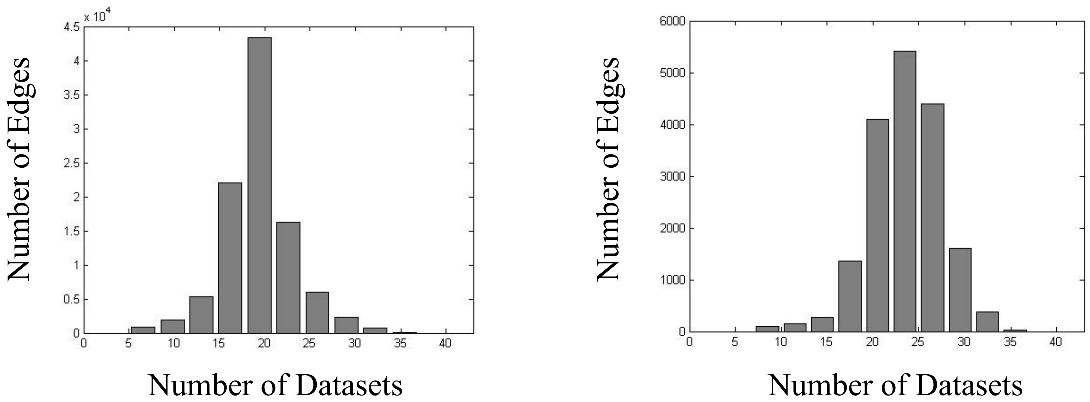


**Distribution of the number of datasets that contribute to each edge**
For each edge connecting a pair of genes in the 0.3 (A) and 0.4 (B) networks, connecting a pair of genes, we calculated the number of datasets in which the two genes appear co-regulated. The data in the histogram shows the number of edges confirmed per number of datasets.
